# Recent Advances in CuInS_2_-Based Photocathodes for Photoelectrochemical H_2_ Evolution

**DOI:** 10.3390/nano13081361

**Published:** 2023-04-14

**Authors:** Noyoung Yoon, Oh Shim Joo, Sang Youn Chae, Eun Duck Park

**Affiliations:** 1 Clean Energy Research Center, Korea Institute of Science and Technology, Seoul 02792, Republic of Korea; 2 Department of Chemical and Biomolecular Engineering, Yonsei University, Seoul 03722, Republic of Korea; 3 Department of Energy Systems Research, Ajou University, Suwon 16499, Republic of Korea; 4 Institute of NT-IT Fusion Technology, Ajou University, Suwon 16499, Republic of Korea; 5 Department of Chemical Engineering, Ajou University, Suwon 16499, Republic of Korea

**Keywords:** copper indium sulfide, photoelectrochemical cell, hydrogen evolution reaction, heterojunction, cocatalyst

## Abstract

Photoelectrochemical (PEC) H_2_ production from water using solar energy is an ideal and environmentally friendly process. CuInS_2_ is a p-type semiconductor that offers many advantages for PEC H_2_ production. Therefore, this review summarizes studies on CuInS_2_-based PEC cells designed for H_2_ production. The theoretical background of PEC H_2_ evolution and properties of the CuInS_2_ semiconductor are initially explored. Subsequently, certain important strategies that have been executed to improve the activity and charge-separation characteristics of CuInS_2_ photoelectrodes are examined; these include CuInS_2_ synthesis methods, nanostructure development, heterojunction construction, and cocatalyst design. This review helps enhance the understanding of state-of-the-art CuInS_2_-based photocathodes to enable the development of superior equivalents for efficient PEC H_2_ production.

## 1. Introduction

The use of renewable energy to reduce greenhouse gas emissions and environmental problems caused by fossil fuels is attracting considerable attention [1]. However, the intermittent nature of renewable energy necessitates the use of diverse energy storage systems. In particular, hydrogen is a promising, environmentally friendly, and carbon-neutral energy resource that emits only water while providing energy [2]. Hydrogen can be utilized in various systems, such as gas turbines, combustors, internal combustion engines, and fuel cells. However, CO_2_-emitting fossil fuels are primarily being used to produce hydrogen owing to the economic feasibility of this strategy, thereby preventing the realization of a carbon-neutral society. In contrast, the production of hydrogen and oxygen through water splitting using renewable solar energy is an ideal method for storing solar energy in chemical form. Photoelectrochemical (PEC) water splitting is a representative method for converting solar light into chemical energy. PEC cells comprise a semiconductor photoelectrode, a counter electrode, and an electrolyte. When photons are absorbed by the photocatalytic electrode, photo-excited electron–hole pairs drive the water-splitting reaction (H_2_O(l) → H_2_(g) + 1/2O_2_(g), $\Delta H_{f,298\;K}^{0}=-285.8\;\mathrm{kJ}/\mathrm{mol}$). However, the commercialization of PEC hydrogen production technology is hindered by its low solar-to-hydrogen conversion efficiency [3]. Therefore, the efficiency of PEC energy conversion must be improved.

A PEC cell is an ideal and environmentally friendly system for producing hydrogen via water photolysis, similar to photosynthesis in nature. Unlike photocatalytic water splitting, PEC water splitting does not require the separation of the generated hydrogen and oxygen. Moreover, PEC cells have theoretically been predicted to achieve high solar-to-hydrogen conversion efficiencies depending on a single photo absorber or tandem configuration system (10–30%) [4]. In particular, the semiconductor photoelectrode is the most important part of PEC cells, as it interacts with photons and converts them into other forms of energy via charge-carrier excitation. Therefore, semiconductor materials suitable for photoelectrodes must be developed to increase the efficiency of the PEC reaction [5].

**Figure 1:**
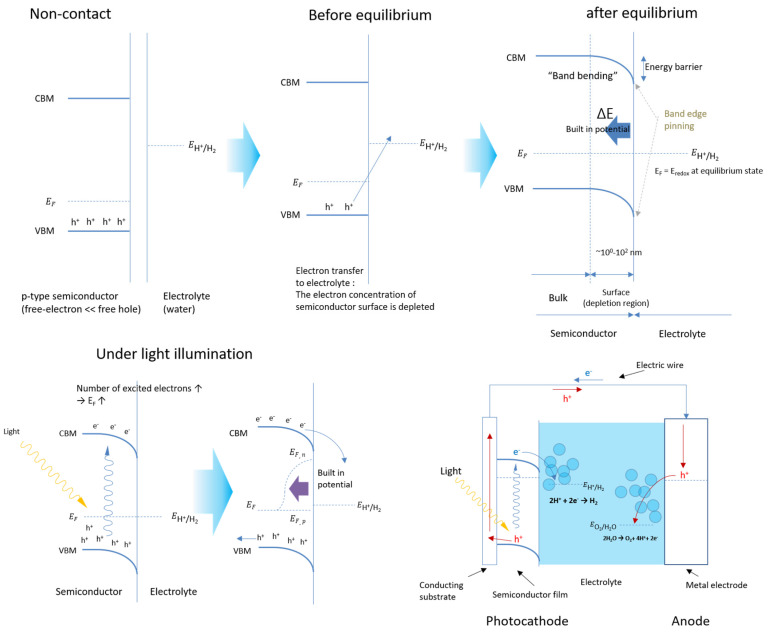
Band bending of p-type semiconductors in electrolytes. When the semiconductor and electrolyte are contacted, the Fermi level of the semiconductor and the chemical potential of the electrolyte become equilibrated and lead to a ‘band-bending energy’ structure. Under the light illumination condition, the excess photo-excited charge carrier breaks the equilibrium state and leads to charge separation via built-in potential at the semiconductor–electrolyte interface. A schematic of a photoelectrochemical (PEC) cell with a p-type semiconductor photocathode is also depicted for water splitting. Photo-excited electron is transported to the semiconductor/electrolyte interface, while the photo-excited hole is transported to the counter electrode through electrical wire and then to the counter electrode–electrolyte interface.

When semiconductor electrodes are in contact with electrolytes, the semiconductor experiences band bending owing to equilibration between the Fermi level of the semiconductor and the redox potential of the liquid. Under this band-bending condition, a depletion layer is formed, and a built-in potential is generated. When a semiconductor–liquid interface is irradiated with light, photons are absorbed by the semiconductor, and excited electron–hole pairs are subsequently generated. The electron–hole pairs are separated by the built-in potential; the electrons reduce the chemical species, and the holes oxidize the chemical species separately. In the case of n-type semiconductors, holes are transported into the semiconductor–liquid interface, producing oxygen, whereas in p-type semiconductors, electrons are transported into the semiconductor–liquid interface, producing hydrogen (Figure 1).

Efficient PEC water-splitting reactions with semiconductor materials can occur under various conditions. First, the bandgap of semiconductor materials should be smaller than 3.0 eV for the efficient utilization of visible solar light. Second, the bandgap must be higher than 1.23 eV to generate a sufficient driving force for the water-splitting reaction. Third, the band position (that is, the energy levels of the conduction and valence bands) should be located at a suitable level compared to the redox potential of water. The conduction band of the semiconductor should have a higher energy level than that of HER, while the valence band of the semiconductor should have a lower energy level than that of OER. Additionally, the semiconductor material should be free from photo-corrosion during the PEC reactions, or the semiconductor should be protected by a passive layer from corrosion [6].

**Figure 2:**
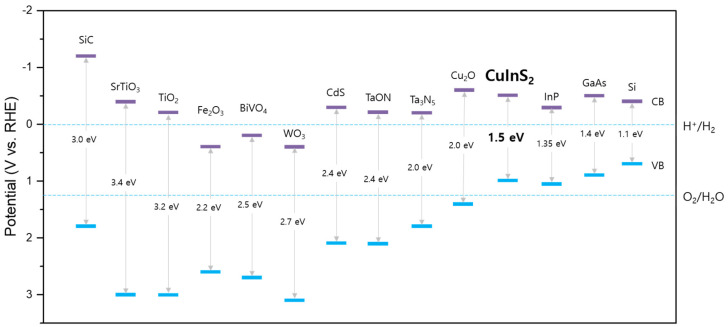
Bandgap and band position of various semiconductors.

Figure 2 shows the bandgaps and band positions of commonly investigated semiconductors. However, semiconductors that can utilize visible solar light, exhibit PEC stability, and spontaneously decompose water without an external potential have not yet been reported. Therefore, various strategies have been attempted to resolve these shortcomings of semiconductor photoelectrodes.

**Figure 3:**
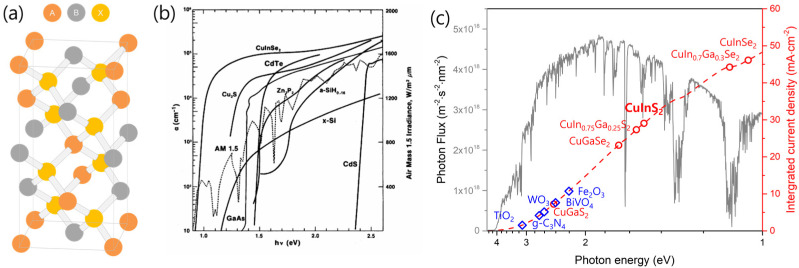
(**a**) Chalcopyrite unit-cell. (**b**) Absorption coefficient of various semiconductor materials [9]. (**c**) Bandgap of chalcopyrite semiconductors and other semiconductor materials. Integrated photocurrent density under 1.5 AM illumination was calculated based on band gap of semiconductors. Reproduced from ref. [9] with permission of the American Physical Society.

Among the reported semiconductor materials, chalcopyrites are attractive photocatalytic substances appropriate for PEC hydrogen production. Chalcopyrites are semiconductors with the chemical composition ABX_2_ and have a high absorption coefficient. Additionally, their band position is suitable for the hydrogen evolution reaction (HER). In particular, Cu-based chalcopyrite composites (A = Cu or Ag; B = In, Ga, or Al; X = S or Se) have a significantly high absorption coefficient (~10^5^ cm^−1^) owing to their direct bandgap structure, resulting in superior light-absorption characteristics compared to those of other semiconductor materials (Figure 3) [7]. Moreover, the bandgap of chalcopyrites can be easily adjusted (1.1–2.5 eV) by forming a solid solution via the replacement of the same-group atoms in the crystal lattice [8]. Furthermore, the higher level of the chalcopyrite conduction band allows excited electrons to have sufficient energy for driving the HER.

In this review, research trends in the use of photocathodes based on CuInS_2_—a noteworthy chalcopyrite semiconductor—for PEC hydrogen evolution are surveyed. CuInS_2_ has attractive properties, such as a suitable band position and bandgap for photocatalytic electrodes. Moreover, it is an economical chalcopyrite because it does not comprise relatively expensive elements, such as Ag, Ga, and Se. Additionally, it can be synthesized using economical methods, such as non-vacuum-based electrodeposition, solvothermal processes, and screen-printing techniques. Although concerns related to indium resource depletion exist, the amount of indium in the Earth’s crust is sufficient for solar energy conversion and industrial applications [10].

The advantageous physical properties of CuInS_2_ permit PEC hydrogen production. Because CuInS_2_ has a bandgap of ~1.5 eV, which enables solar light absorption over an extremely wide range (from ultraviolet to near-infrared) and a high extinction coefficient (>10^5^ cm^−1^) [11], it has been used in solar energy conversion systems, such as solar cells and photocatalysts, for a long time [12,13,14,15]. Although CuInS_2_ generally exhibits p-type semiconductor characteristics, it can also attain n-type semiconductor attributes through the control of Cu vacancies or dopants [16,17,18]. However, in most studies on PEC cells, CuInS_2_ is used as a p-type semiconductor and as a photocathode for hydrogen evolution. Despite having an excellent light-absorption ability, CuInS_2_-based photoelectrodes generate a photocurrent of ~16 mA∙cm^−2^, which is approximately half of the theoretical photocurrent (~28.96 mA∙cm^−2^) predicted from the bandgap (1.55 eV). Table 1 summarizes the activity of CuInS_2_ photocathodes reported to date for PEC hydrogen evolution in terms of the photocurrent density, onset potential, and half-cell applied-bias photon-to-current conversion efficiency (HC-ABPE). The activity of CuInS_2_ remains restricted by the high recombination of photogenerated electron–hole pairs, a surface state, inferior electrochemical catalytic properties, and low photochemical stability [19,20].

Therefore, methods for fabricating CuInS_2_ photocathodes and strategies for improving their PEC performance are summarized and discussed in this review.

## 2. Synthesis Methods and Morphological Control Techniques for CuInS_2_ Photocathodes

**Figure 4:**
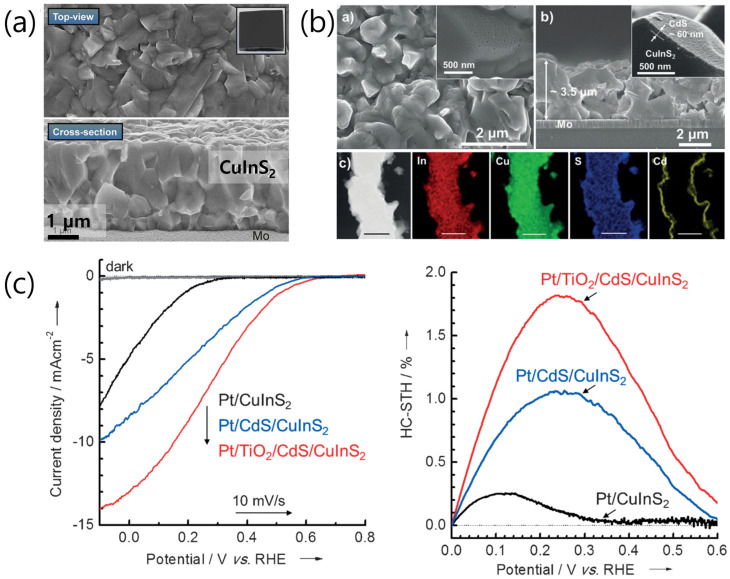
Crystal morphology of CuInS_2_ thin films prepared by (**a**) sputtering [33] and (**b**) electrodeposition/sulfurization [42]. (**c**) Linear sweep voltammetry (LSV) profiles and HC-ABPE data of CuInS_2_-based photocathodes fabricated by electrodeposition/sulfurization [42]. Reproduced from ref. [29] with permission of the Royal Society of Chemistry. Reproduced from ref. [39] with permission from Wiley-VCH.

The synthesis methods for CuInS_2_ thin films can be categorized into physical and chemical techniques. Sputtering and co-evaporation are representative physical methods that require ultrahigh-vacuum conditions, under which phase-pure chalcopyrite crystals with minimal impurities can be grown. Sputtering is a typical method for synthesizing CuInS_2_ under vacuum conditions, in which Cu and In are sequentially deposited on substrates and converted into CuInS_2_ through sulfurization. This method can produce thin films with excellent uniformity and readily controllable stoichiometries. However, the stipulated vacuum conditions lead to a remarkably high thin-film fabrication cost, increasing the unit cost of hydrogen production [52]. Therefore, wet chemical methods are being studied as an alternative CuInS_2_ synthesis strategy (Figure 4).

**Figure 5:**
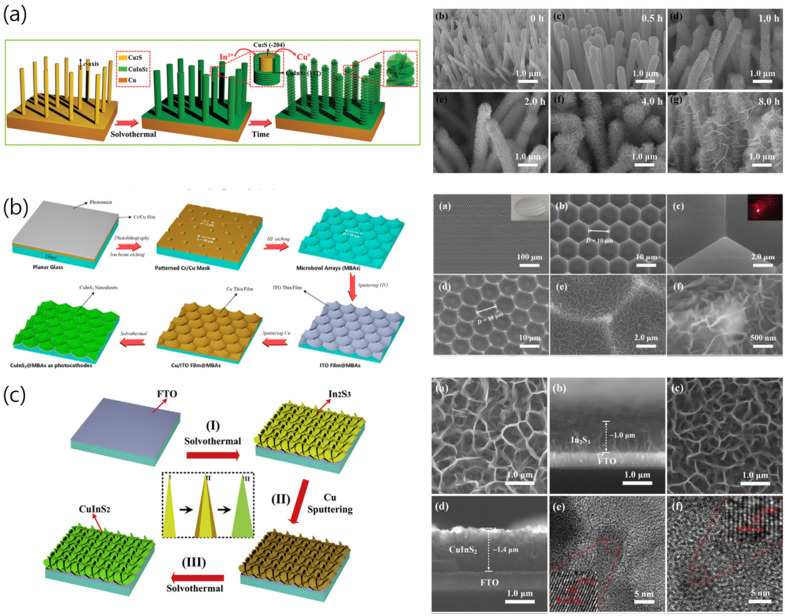
Schematics illustrating the preparation of various nanostructure-controlled CuInS_2_ films and scanning electron microscopy (SEM) images of the films: (**a**) CuInS_2_ nanorods array by facile gas–solid reaction/solvothermal [28], (**b**) photoelectrode based on 3D micro bowl arrays using CuInS_2_ nanosheet array grown using solvothermal method as photocatalyst [26], and (**c**) CuInS_2_ nanosheet arrays fabricated from In_2_S_3_ self-sacrificial templates using solvothermal and sputtering [25]. Reproduced from ref. [28] and ref. [26] with permission from Elsevier. Reproduced from ref. [25] with permission from Wiley-VCH.

CuInS_2_ can also be synthesized using solution-based approaches, such as chemical bath deposition, electrodeposition, spin coating, spray pyrolysis, and hydrothermal methods, which facilitate economical and large-scale preparation. Spin coating involves coating Cu/In precursor ink onto a rotating substrate. In this method, the Cu/In ratio can be easily controlled by adjusting the composition of the precursor solution; moreover, the thin-film thickness can be regulated by repetitive spin coating [53]. Spray pyrolysis, which involves spraying a precursor solution onto a high-temperature substrate, facilitates the preparation of large-area films. Here, the CuInS_2_ composition can be adjusted using the concentration of the spray solution [23]. Electrodeposition–sulfurization is predominantly used to synthesize CuInS_2_ photocathodes for PEC hydrogen evolution. Here, Cu and In are electrochemically deposited on conductive substrates in an electrolytic solution [42]. Co-deposition has also been performed to fabricate Cu–In alloy films [21]. Simultaneous electrodeposition of three elements can be performed in a Cu–In–S ternary electrolytic bath. Unlike other chalcopyrite materials, the CuInS_2_ prepared by electrodeposition exhibits similar or even higher performance than that of the CuInS_2_ photoelectrodes prepared by co-evaporation or sputtering. Therefore, electrodeposition is a promising technique for synthesizing high-performance CuInS_2_ thin films (Figure 5).

However, the aforementioned methods are limited in terms of permitting nanostructural control to achieve high PEC activities. The multidimensional structure of semiconductor thin films is often critical to determining the PEC activity. One-dimensional (1D) nanostructures allow efficient PEC reactions owing to the improved charge separation/transport, and their unique optical and electrical properties lead to enhanced light absorption [31]. Additionally, most nanostructures exhibit higher light-trapping efficiencies and photocurrent densities than those of thin films of various materials. For example, photoelectrodes with structures such as nanowire arrays (NWAs), nanorod arrays, and nanotubes have the advantage of charge separation owing to the improved charge-carrier collection, rapid minority-carrier diffusion, and robustness of the electrode–electrolyte interface [31].

Three-dimensional branched nanoarray structures can improve the PEC performance of CuInS_2_ photocathodes by enhancing their light-absorption characteristics and surface area. Moreover, two-dimensional structures can improve the PEC performance through enhancements in the electronic and optical properties and surface area. In particular, layered bonding is beneficial in terms of promoting photocarrier separation and improving the PEC performance by shortening the charge-transport duration and distance [28]. However, the complex nature of multinary chalcogenide materials hinders the development of practical techniques for synthesizing vertically aligned structures. Most previously reported copper chalcopyrite nanostructures have been synthesized using hard nanoscale templates, such as anodic aluminum oxide (AAO). Yang et al. prepared CuInS_2_ photocathodes with nanorod structures by impregnating AAO templates with a Cu/In precursor solution and used them for PEC hydrogen production [31]. The charge-carrier collection was found to be inefficient when the nanowire length was greater than the carrier-diffusion length (~1 μm). Additionally, the transport of photogenerated electrons to carrier collectors was suppressed as the surface area of CuInS_2_ decreased with increasing nanowire diameter. Therefore, the 1D nanostructures had to be optimized in terms of diameter and length to maximize their advantages. Moreover, the control of the pores and depth of AAO templates have been reported to be key to preparing CuInS_2_ nanorod arrays of an appropriate length. Hydrothermal/solvothermal approaches have also been implemented to adjust the CuInS_2_ nanostructures [24,25,36]. Tu et al. deposited In_2_S_3_ nanosheet arrays on a fluorine-doped tin oxide (FTO) substrate using a solvothermal method and used them as self-sacrificing templates. Substantial CuInS_2_ deposition was performed on the 2D structure, yielding 2D CuInS_2_ nanosheets exhibiting strong excitonic effects, which led to an enhanced optoelectronic response and reduced radiative lifetime. This nanosheet array has the advantages of improved light collection and photogenerated charge pair creation [25].

## 3. Heterojunction Interface for CuInS_2_

The low photovoltage generated by the rapid recombination of electron–hole pairs is a major factor that impedes photoelectrode activity [20]. In particular, the rate of hydrogen generation from CuInS_2_ is significantly reduced, owing to its low photovoltage. However, the hydrogen production efficiency can be improved by creating a p–n junction between p-type CuInS_2_ and n-type semiconductors [54].

Two types of p–n junctions exist—homojunctions and heterojunctions—which comprise the same and two different semiconductor materials, respectively. As they permit adjustment of the energy level that is matched with the bandgap between CuInS_2_ and other semiconductor materials, p–n-type heterojunctions have been extensively used to suppress the recombination of electron–hole pairs in CuInS_2_ photocathodes [34]. Among them, CdS is predominantly used for chalcopyrites and different p-type photoelectrodes (p-SnS, CdTe, and CuZnSnS_4_) and is known to significantly improve photovoltage [54].

**Figure 6:**
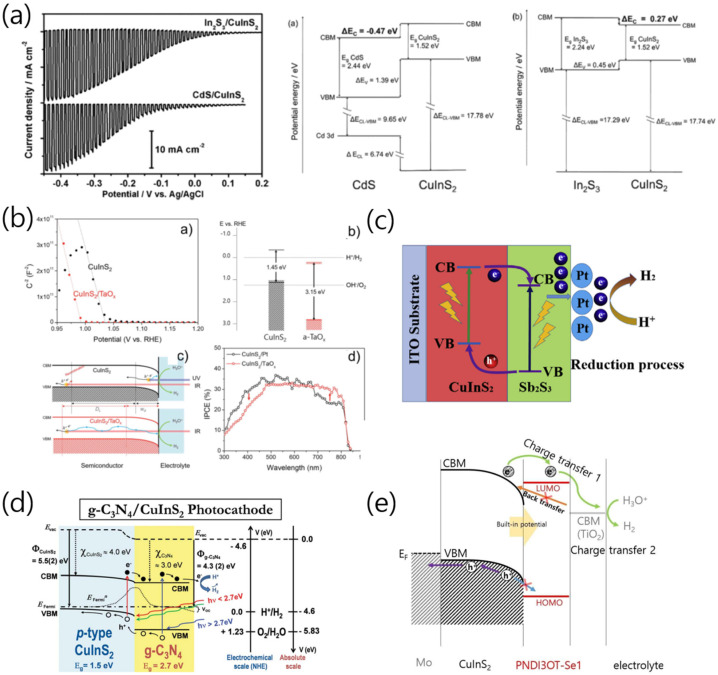
(**a**) Under chopped illumination of simulated sunlight (AM1.5), LSV profiles and difference in energy levels between CdS and In_2_S_3_ upon formation of a heterojunction with CuInS_2_ for CuInS_2_/CdS and CuInS_2_/In_2_S_3_ photocathodes using an aqueous solution 0.2 M Eu(NO_3_)_3_ (pH 4) [39]. (**b**) Mott–Schottky plots of CuInS_2_ and CuInS_2_/TaO_x_, Fermi-level shift of the CuInS_2_/TaO_x_ heterojunction, a schematic diagram of band bending of CuInS_2_ and CuInS_2_/TaO_x_ photoelectrodes and incident-photon-to-electron conversion efficiency (IPCE) values for CuInS_2_/TaO_x_ photocathodes [20]. (**c**) Schematic diagram of the CuInS_2_/Sb_2_S_3_/Pt photoelectrodes. This band alignment indicates the charge-transfer process [27]. (**d**) Proposed energy band diagram of the CuInS_2_/C_3_N_4_ [33]. (**e**) Schematic energy diagrams of CuInS_2_/PNDI3OT-Se1-polymer heterojunctions, indicating charge transfer between semiconductors [48]. Reproduced from ref. [39] with permission from the American Chemical Society. Reproduced from ref. [20,33,48] with permission from the Royal Society of Chemistry. Reproduced from ref. [27] with permission from Elsevier.

The difference in energy levels between two materials is particularly critical for enabling charge transfer at a heterojunction interface. The CuInS_2_/CdS interface has a cliff-type heterojunction with a difference between conduction band levels of −0.47 eV. When excited electrons move from CuInS_2_ to CdS through the conduction band, the energy difference (1.05 eV) between the conduction band of CdS and the valence band of CuInS_2_ increases the recombination chance of electron–hole pairs at the interface. However, the CuInS_2_–In_2_S_3_ interface has a notch-type energy structure when the heterojunction is formed. Moreover, the energy difference (1.79 eV) between the conduction band of In_2_S_3_ and the valence band of CuInS_2_ is relatively larger than that of CuInS_2_/CdS. Therefore, the electron–hole recombination is suppressed, and charges can be efficiently separated and transported to the reactant [39]. Owing to this difference in the energy structure, the CuInS_2_/In_2_S_3_ photoelectrode (−18 mA∙cm^−2^) generates a higher photocurrent than that of CuInS_2_/CdS (Figure 6). 

MoS_x_, Ni-MoS_x_, SnS_2_, Sb_2_S_3_, and TaO_x_ can also be used as n-type semiconductors for heterojunction formation with CuInS_2_ photocathodes. MoS_x_ electrodeposited on CuInS_2_ was found to generate an additional photovoltage of ~300 mV owing to p–n junction formation [35]. Moreover, Chae et al. found that the in situ transformation of crystalline MoS_x_ into amorphous MoS_x_ enhanced the charge transfer at the MoS_x_/CuInS_2_ interface, which improved the PEC HER activity [47]. Zhao et al. reported that Ni doping of MoS_x_ increased the PEC activity owing to the improved hydrogen evolution kinetics of MoS_x_. The photocathode with the Ni-MoS_x_/CuInS_2_ heterojunction structure exhibited a higher onset potential (0.5 V_RHE_) than that of bare CuInS_2_ photocathodes [34].

The introduction of n-SnS_2_ with a bandgap of 1.62 eV into CuInS_2_ photoelectrodes can permit effective charge transfer owing to the energy-level difference between the two materials [36]. Similarly, at the interface between CuInS_2_ and TaO_x_, which is a multifunctional passive layer with lower conduction and valence bands than those of CuInS_2_, electrons and holes are efficiently transferred by a cliff-like energy-level structure similar to that of the CuInS_2_/SnS_2_ heterojunction. In particular, TaO_x_ has been observed to act as a multifunctional passivation layer that not only suppresses the recombination of electron–hole pairs but also increases the activity and stability for hydrogen production. Therefore, the incident-photon-to-electron conversion efficiency (IPCE) has been found to increase in longer-wavelength regions owing to the prolonged charge-carrier lifetime, resulting in a three-fold higher photocurrent than that of the CuInS_2_ photoelectrodes [4].

Recently, atomically graded passivation layers were developed for CuInS_2_ photocathodes. First, Mo:TaO_x_ layers were prepared by implanting Mo atoms into TaO_x_ layers. The resulting Mo:TaO_x_ film was then sulfurized, yielding a (Ta,Mo)_x_(O,S)_y_ layer. The graded distribution of Mo and S in the TaO_x_ matrix led to the synergetic PEC activity of CuInS_2_, similar to that of CuInS_2_ photocathodes decorated with Pt cocatalysts [32]. n-Type polymer semiconductors could also be used to form p–n heterojunctions with CuInS_2_. When the n-type polymer semiconductor PNDI3OT-Se was deposited on CuInS_2_, the onset potential shifted, and the photocurrent increased, similar to that in an inorganic p–n junction, such as the CdS/CuInS_2_ heterojunction [48]. The band position of PNDI3OT was adjusted using the monomer structure. Moreover, the valence band position of the polymer and CuInS_2_ were critical for enabling efficient charge transfer at the polymer/CuInS_2_ interface. This result highlights the potential of polymer/inorganic hybrid photoelectrodes for realizing efficient hydrogen production.

## 4. Cocatalyst Decoration for Hydrogen Evolution

**Figure 7:**
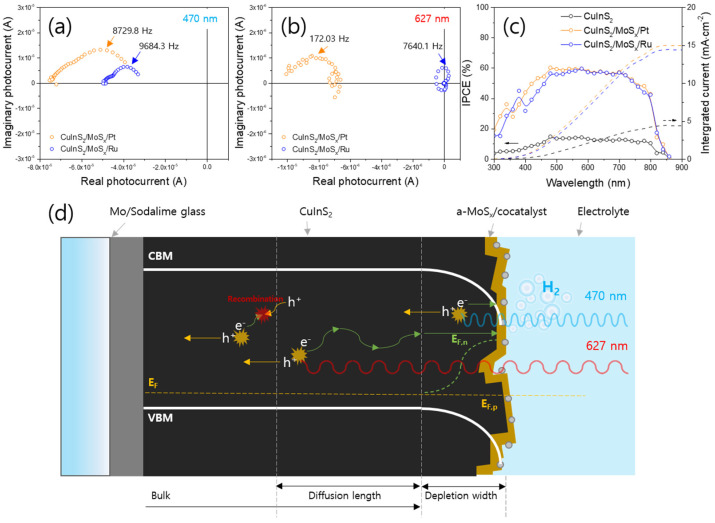
Effects of Ru and Pt cocatalysts on CuInS_2_/MoS_x_ photocathodes, such as the improved transient time of electrons by the Ru cocatalysts. Intensity-modulated photocurrent spectroscopy results of CuInS_2_-based photocathodes under (**a**) 470 nm and (**b**) 627 nm LED light. (**c**) IPCE data of CuInS_2_-based photocathodes. (**d**) Schematic diagram of charge carrier transportation at CuInS_2_/MoS_x_/cocatalysts interfaces [35]. Reproduced from ref. [31] with permission from Elsevier.

Even if photogenerated charges have a sufficient thermodynamic potential, they can easily recombine if the HER on the photoelectrode surface is kinetically unfavored. Poor kinetics at the semiconductor surface leads to a high overpotential; however, cocatalysts can reduce the activation energy, boosting the HER rate. Therefore, the HER rate of photocathodes can be improved by applying suitable cocatalysts onto the surface of semiconductor photoelectrodes. Noble metals are considered excellent electrocatalysts because of their high conductivity and low overpotential for the HER. Among noble and transition metals, Pt is known to be one of the best electrocatalysts for the HER. The activity trends can be explained using a ‘volcano plot’ showing the adsorption/desorption energy of hydrogen ions on the catalyst surface. Therefore, noble metals are extensively used as cocatalysts for photocathodes; however, their expensiveness necessitates the development of economical cocatalyst alternatives. For instance, relatively inexpensive transition metal cocatalysts or protective layers have been used to enhance the activity and stability of Cu(In,Ga)Se_2_ (CIGS) photoelectrodes. Moreover, Ni-Mo cocatalysts have improved the stability and activity of CIGS photoelectrodes under neutral electrolyte conditions [55]. When a Ti-Mo layer was introduced between Pt cocatalysts and CIGS/CdS electrodes, the stability and activity were significantly increased in neutral electrolytes [56]. However, several transition metals are unstable under low-pH conditions, which limits the use of strongly acidic electrolytes. Transition metal–sulfide cocatalysts (MoS_x_, CoS_x_, CoSe_x_, and NiS_x_) have exhibited high hydrogen-generation activity in chalcopyrite and Si photoelectrodes [57,58,59,60,61,62]. However, the development of cocatalysts for CuInS_2_ photoelectrodes is lagging behind that of other chalcopyrite-based photoelectrodes, with Pt cocatalysts being used in most cases. Therefore, various HER cocatalysts must be applied to CuInS_2_-based photoelectrodes, and their activity and mechanism should be investigated (Figure 7).

Charge transfer at the photoelectrode–cocatalyst interface should also be the focus, along with the electrocatalytic activity of cocatalysts. Although Pt is experimentally and theoretically regarded as one of the best HER electrocatalysts, it may be unsuitable for application on photoelectrode surfaces. Patra et al. used Au nanoparticles as a cocatalyst with CuInS_2_ particles, which significantly increased the hydrogen evolution activity owing to the smooth charge transport between CuInS_2_ and Au through surface-Plasmon-resonance effects [37]. Chae et al. incorporated photoelectrodes with Ru, which has a lower production activity than that of Pt and achieved a high solar-to-hydrogen conversion efficiency [35]. The high work function of Ru induced effective charge transfer from CuInS_2_/MoS_x_ along with ohmic junction formation, whereas the CuInS_2_/MoS_x_/Pt interface did not form an ohmic junction. Therefore, charge transfer at the cocatalyst–CuInS_2_ interface must be considered for designing efficient cocatalysts.

## 5. Hole Transport Layers

Generally, holes in the p-type chalcopyrite semiconductor CuInS_2_—which are a majority carrier—have higher mobility and longer lifetimes than those of electrons [63]. Therefore, improving the electron transport/transfer in the semiconductor layer or at the semiconductor–electrolyte interface has received more attention than hole transportation. However, the enhancement of hole transfer has been found to be crucial for improving the activity of CuInS_2_. In most cases, CuInS_2_ crystals have been grown on Mo foil or Mo-coated substrates because of the ohmic contact enabled between Mo and CuInS_2_ (or other chalcopyrite materials) [64,65]. However, transparent substrates can be effective depending on the direction of illumination for determining the charge-transport property of photoelectrodes [66]. CuInS_2_ films have been grown on transparent conductive substrates, such as glass coated with indium tin oxide (ITO) or fluorine-doped tin oxide (FTO), which have been extensively used for PEC cells. Liu et al. reported that FeOOH interlayers between CuInS_2_ and ITO improved the hole transfer from CuInS_2_ to the ITO layer, which led to the FTO/FeOOH/CuInS_2_/Pt photocathodes exhibiting a photoactivity 3.1 times higher than that of ITO/CuInS_2_ photocathodes [50]. FeOOH effectively captured excited holes owing to its hole-storing tendency, and the electron–hole pair recombination of CuInS_2_ was suppressed. Kumar et al. synthesized NaNbO_3_/CuInS_2_/In_2_S_3_ and NaNbO_3_/In_2_S_3_/CuInS_2_ core/shell/shell nanocubes for PEC applications [49]. In the NaNbO_3_/CuInS_2_/In_2_S_3_ system, CuInS_2_ and In_2_S_3_ exhibited z-scheme behavior; however, NaNbO_3_/In_2_S_3_/ CuInS_2_ had a type II band structure. The z-scheme nanocube immobilized on ITO exhibited a considerably higher photocurrent than that of the electrodes immobilized with the type-II-structured nanocube. This type of energy-level design may facilitate hole transfer at a non-ohmic interface between CuInS_2_ and the transparent, conductive metal-oxide layer (ITO or FTO).

## 6. Solid Solutions of CuInS_2_

Solid solutions of chalcopyrite CuInS_2_ crystals and other atoms can be readily prepared. For example, various photocathode materials, such as Cu(In,Ga)Se_2_, CuGaSe_2_, (Ag,Cu)GaSe_2_, and CuIn(S,Se)_2_, have been studied for the HER [67,68,69,70]. However, the activity and physicochemical properties of this type of material are beyond the scope of this review. Nevertheless, the solid solution of chalcopyrite CuInS_2_ and zincblende ZnS is worth mentioning. Zhao et al. prepared solid solution crystals of (CuInS_2_)_x_(ZnS)_1-x_ using an electrodeposition–sulfurization method [41]. The enlarged bandgap of the solid solution (CuInS_2_)_x_(ZnS)_1-x_ led to a significantly high onset potential (up to 0.8 V_RHE_). The half-cell solar-to-hydrogen conversion efficiency of the (CuInS_2_)_x_(ZnS)_1-x_/CdS/Pt photocathodes was 5.6%, which is a remarkably high value among CuInS_2_-based photocathodes. This result emphasizes the benefits of solid solutions containing CuInS_2_ or other materials over those of the simple bilayer-structured equivalents because of the graded band structure obtained by the atomic gradation of the semiconductor.

## 7. Summary and Outlook

CuInS_2_-based photocathodes for PEC hydrogen evolution are reviewed herein, with a focus on their synthesis methods, heterolayer construction, and cocatalyst design. CuInS_2_ has been synthesized using physical (co-evaporation and sputtering) and chemical techniques (electrodeposition, spin coating, spray pyrolysis, and hydrothermal methods). Generally, the physical methods yield a dense, high-quality chalcopyrite thin film with large grains and high crystallinity, with the highly crystalline semiconductors exhibiting high PEC activity. Therefore, the physical methods enable higher PEC performance than that of the solution-based methods. However, unlike other quaternary or pentanary chalcopyrite materials, CuInS_2_ synthesized using electrodeposition methods has similar or superior PEC activity compared to that of CuInS_2_ films prepared using physical methods. Additionally, certain solution-based methods offer advantages in controlling the morphological features of CuInS_2_, such as dimensions and porosity. However, the complex nanostructure can also have more defect sites than those in a highly crystallized thin film. The activity of CuInS_2_-based photoelectrodes synthesized to date using solvo(hydro)thermal or other solution-based methods is considerably inferior to that of specimens prepared by electrodeposition. Therefore, nanostructural control remains insufficient for overcoming the rapid charge recombination at the defect or surface states of the CuInS_2_ crystal. A new solution-based synthesis method for CuInS_2_ with high PEC activity remains to be developed.

The use of heterojunctions to suppress electron–hole recombination has been extensively studied. Many sulfide materials (CdS, In_2_S_3_, ZnS, NiS_x_, and MoS_x_) have been investigated for constructing a heterojunction or p–n junction with CuInS_2_. Moreover, certain metal oxides, atomic-graded metal oxides, and n-type polymer semiconductors have also been introduced. The bandgap alignment between CuInS_2_ and heteromaterials is critical to enabling efficient charge transfer at the interface. Suitable band energy matching can successfully suppress the electron–hole recombination, boosting the photocurrent and improving the onset potential.

Cocatalysts that can facilitate hydrogen evolution from a photoelectrode surface are also important. The CuInS_2_ surface is kinetically unfavorable for the HER and thereby requires a cocatalyst to reduce the overpotential. Pt, which has been widely used as a cocatalyst for CuInS_2_-based photocathodes, should be replaced with economical and earth-abundant materials. Although certain noble metals and metal sulfides have been investigated as cocatalysts for CuInS_2_ photoelectrodes, more diverse cocatalyst materials should be examined. Additionally, when a cocatalyst is applied to a PEC system, the electrochemical activity of the cocatalyst, as well as the interfacial charge transfer between CuInS_2_ and the cocatalyst, must be considered. Similarly, interfacial charge transfer between CuInS_2_ and substrates can be controlled using hole-transporting interlayers. Finally, the use of a partial solid solution at the interface with CuInS_2_ can be an effective strategy for enabling efficient charge transfer at several interfaces in a photoelectrode structure. All these strategies should be combined to achieve the theoretical limit of a PEC cell with a CuInS_2_ photoelectrode.

## Figures and Tables

**Table 1:** Activity of CuInS_2_-based photocathodes for PEC hydrogen evolution.

Photoelectrode	Substrate	Film Morphology	CuInS_2_ Synthesis Method	Current Density (mA∙cm^−2^)	Onset Potential (V_RHE_)	HC-ABPE ^1^ (%)	Electrolyte	Ref.
CuInS_2_	Mo foil	Thin film	Co-electrodeposition/sulfurization	−0.75	0.18	-	0.1 M Eu(NO_3_)_3_ (pH 2.3)	[21]
CuInS_2_	Mo/glass	Thin film	Electrodeposition/sulfurization	−7 at −0.4 V_Ag/AgCl_	−0.2 V_Ag/AgCl_	-	0.2 M Eu(NO_3_)_3_	[22]
CuInS_2_	Mo/glass	Thin film	Spray pyrolysis/sulfurization	−2.3 at −0.4 V_Ag/AgCl_	0.15 V_Ag/AgCl_	-	0.1 M Eu(NO_3_)_3_ (pH 4)	[23]
CuInS_2_	ITO/glass ^2^	Network-like nanostructure	Hydrothermal method	−0.4 at −0.4 V_RHE_	0.1	-	0.1 M Na_2_SO_4_ (pH 6.7)	[24]
CuInS_2_	FTO/glass ^3^	Nanosheet arrays	Solvothermal/sputtering	−0.05	0.5	-	1.0 M KCl	[25]
CuInS_2_	ITO/glass	Nanosheet	Solvothermal	−0.07	−0.05	-	1.0 M KCl (pH 5.97)	[26]
CuInS_2_	ITO/glass	Heterostructure	Hydrothermal	−0.2 at −0.4 V_RHE_	0	-	01 M Na_2_SO_4_	[27]
CuInS_2_	Cu foil	Nanowire arrays	Facile gas–solid reaction/solvothermal	−0.1	0.2	-	1.0 M KCl (pH 5.97)	[28]
CuInS_2_/CdS	Cu foil	Nanowire arrays	Facile gas–solid reaction/solvothermal	−0.48	0.2	-	1.0 M KCl (pH 5.97).	[28]
CuInS_2_/CdS	Cu mesh	Nanowire arrays	Gas–solid reaction/solvothermal	−0.3	0.05	-	1.0 M KCl (pH 5.97)	[29]
CuInS_2_/CdS	FTO/glass	Nanocrystalline	Spin coating	−1.2	0.49	-	20 mM MV^2+^ 0.5 M Na_2_SO_4_	[30]
CuInS_2_/CdS/ZnS	Glass	Nanorod arrays	AAO template growth/sulfurization ^4^	−0.01	0.3	-	0.5 M Na_2_SO_4_ (pH 10)	[31]
CuInS_2_/CdS/(Ta,Mo)_x_,(O,S)_y_	Mo/glass	Thin film	Electrodeposition/sulfurization	−12	0.55	-	0.1 M HClO_4_ (pH 1.07)	[32]
CuInS_2_/g-C_3_N_4_	Mo/glass	Thin film	Sputtering/sulfurization	−0.02	0.15	-	0.1 M H_2_SO_4_ (pH 1)	[33]
CuInS_2_/Ni-MoSx	Mo/glass	Thin film	Electrodeposition/sulfurization	−9	0.4	0.68	0.5 M KP_i_ (pH 7)	[34]
CuInS_2_/MoS_x_	Mo/glass	Thin film	Electrodeposition/sulfurization	−9.99	0.36	0.9	0.1 M HclO4 (pH 0.92–0.96)	[35]
CuInS_2_/SnS_2_.	FTO/glass	Nanosheet	Hydrothermal	−1 at −0.45V_RHE_	-	-	0.5 M Na_2_SO_4_	[36]
CuInS_2_/SnS_2_/C_60_	FTO/glass	Nanosheet	Hydrothermal	−1.5 at −0.45V_RHE_	-	-	0.5 M Na_2_SO_4_	[36]
CuInS_2_/TaO_x_	Mo/glass	Thin film	Electrodeposition/sulfurization	−9	0.2	-	0.5 M H_2_SO_4_ (pH 0.35–39)	[20]
CuInS_2_/Pt	Mo/glass	Thin film	Electrodeposition/sulfurization	−9.2	0.24	0.48	0.5 M KP_i_ (pH 7)	[34]
CuInS_2_/Pt	Mo/glass	Thin film	Electrodeposition/sulfurization	−10.97	0.27	0.49	0.1 M HClO_4_ (pH 0.92–0.96)	[35]
CuInS_2_/Ru	Mo/glass	Thin film	Electrodeposition/sulfurization	−8.02	0.15	0.25	0.1 M HClO_4_ (pH 0.92–0.96)	[35]
CuInS_2_/Au	ITO/glass	Dotted disk	Wet chemical synthesis	−12	0.67	4.29	0.5 M Na_2_SO_4_	[37]
CuInS_2_/CdS-Pt	Mo/glass	Thin film	Electrodeposition/sulfurization	−15.12	0.5	1.6	0.1 M Na_2_SO_4_ (pH 9)	[38]
CuInS_2_/CdS-Pt	Mo/glass	Thin film	Electrodeposition/sulfurization	−11	0.55	1.8	0.2 M NaH_2_PO_4_ (pH 6)	[39]
CuInS_2_/CdS/Pt	Mo foil	Thin film	Electrodeposition/sulfurization	−7.5	0.6	-	0.2 M KH_2_PO_4_-Ar (pH 6.0)	[40]
CuInS_2_/CdS/Pt	Mo/glass	Thin film	Electrodeposition/sulfurization	−13.4	0.58	2.3	0.5 M KP_i_ (pH 7)	[41]
CuInS_2_/CdS/Pt	Mo/glass	Thin film	Electrodeposition/sulfurization	−8	0.5	1.06	0.1 M Na_2_HPO_4_ (pH 10)	[42]
CuInS_2_/CdS/TiO_2_/Pt	Mo/glass	Thin film	Electrodeposition/sulfurization	−13	0.6	1.82	0.1 M Na_2_HPO_4_ (pH 10)	[42]
CuInS_2_/CdS/TaO_x_/Pt	Mo/glass	Thin film	Electrodeposition/sulfurization	−16	0.55	-	0.1M HclO_4_ (pH 1.07)	[32]
CuInS_2_/CdS/AZO/TiO_2_/Pt	FTO/glass	Thin film	Electrodeposition/solvothermal	−2	0.4	-	0.5 M Na_2_SO_4_ 0.1 M KH_2_PO_4_ (pH 5.0)	[43]
CuInS_2_/In_2_S_3_-Pt	Mo/glass	Thin film	Electrodeposition/sulfurization	−15.16	0.6	1.9	0.1 M Na_2_SO_4_ (pH 9)	[38]
CuInS_2_/In_2_S_3_-Pt	Mo/glass	Thin film	Electrodeposition/sulfurization	−18	0.65	2.9	0.2 M NaH_2_PO_4_ (pH 6)	[39]
CuInS_2_/In_2_S_3_/Pt	Mo/glass	Thin film	Electrodeposition/sulfurization	−5.6	0.7	0.7	0.1 M Na_2_SO_4_ (pH 10)	[44]
CuInS_2_/In_2_S_3_/Pt	Mo/glass	Thin film	Co-electrodeposition/sulfurization	−5.2	0.5	0.83	0.25 M KH_2_PO_4_ 0.25 M K_2_HPO_4_ (pH 7)	[45]
CuInS_2_/In_2_S_3_/Pt	FTO/glass	Thin film	Electrodeposition/sulfurization	−15	0.78	1.97	0.1 M Na_2_SO_4_ (pH 9)	[46]
CuInS_2_/Sb_2_S_3_/Pt	ITO/glass	Heterostructure	Hydrothermal	−0.2	0.4	-	01 M Na_2_SO_4_	[27]
CuInS_2_/MoS_x_/Ru	Mo/glass	Thin film	Electrodeposition/sulfurization	−12.87	0.34	1.23	0.1 M HclO4 (pH 0.92–0.96)	[35]
CuInS_2_/MoS_x_/Pt	Mo/glass	Thin film	Electrodeposition/sulfurization	−14.82	0.33	1.08	0.1 M HClO_4_ (pH 0.92–0.96)	[35]
CuInS_2_/Ni-MoS_x_/Pt	Mo/glass	Thin film	Electrodeposition/sulfurization	−15.5	0.5	1.48	0.5 M KP_i_ (pH 7)	[34]
CuInS_2_/MoS_x_O_y_	Mo/glass	Thin film	Electrodeposition/sulfurization	−5.94	0.24	-	0.1 M HClO_4_ (pH 0.9–1.0)	[47]
CuInS_2_/PNDI3OT-Se1/TiO_2_/Pt	Mo/glass	Thin film	Electrodeposition/sulfurization	−15.67	0.25	1	0.1 M HClO_4_ (pH 0.9–1.0)	[48]
CuInS_2_/PNDI3OT-Se_2_/TiO_2_/Pt	Mo/glass	Thin film	Electrodeposition/sulfurization	−4.91	0.15	0.15	0.1 M HClO_4_ (pH 0.9–1.0)	[48]
NaNbO_3_/CuInS_2_	ITO/glass	Nanocube core/shell	Hydrothermal method	1.05 at −1 V_Ag/AgCl_	−0.11 V_Ag/AgCl_	-	0.5 M Na_2_SO_4_	[49]
NaNbO_3_/In_2_S_3_/CuInS_2_	ITO/glass	Nanocube core/shell	Hydrothermal method	1.63 at −1 V_Ag/AgCl_	−0.11 V_Ag/AgCl_	-	0.5 M Na_2_SO_4_	[49]
NaNbO_3_/CuInS_2_/In_2_S_3_	ITO/glass	Nanocube core/shell	Hydrothermal method	6.72 at −1 V_Ag/AgCl_	−0.11 V_Ag/AgCl_	-	0.5 M Na_2_SO_4_	[49]
FeOOH/CuInS_2_/Pt	ITO/glass	Nanoparticles	Successive ionic layer adsorption and reaction (SILAR)	−2	0.6	-	01 M Na_2_SO_4_ (pH 7.1)	[50]
NiO/CuInS_2_	ITO/glass	Nanosheets	Hydrothermal	−0.23	0.2	-	01 M Na_2_SO_4_	[51]
NiO/CuInS_2_/NiS	ITO/glass	Nanosheets	Facial hydrothermal	−0.27	0.4	-	01 M Na_2_SO_4_	[51]
(CuInS_2_)_0.81_(ZnS)_0.19_/CdS/Pt	Mo/glass		Electrodeposition/sulfurization	−16.7	0.84	5.6	0.5 M KPi (pH 7)	[41]

^1^ HC-APBE: half-cell applied-bias photon-to-current conversion efficiency; ^2^ ITO: indium tin oxide; ^3^ FTO: fluorine-doped tin oxide; ^4^ AAO: anodized aluminum oxide.

## Data Availability

The data presented in this study are available in this article.
